# Glaucoma spectrum findings among the Yanomami Indigenous people of the Brazilian Amazon

**DOI:** 10.1007/s10792-026-04234-0

**Published:** 2026-09-02

**Authors:** Maria Christina Chagas Ferreira, Marcos Antonio Pellegrini, Bianca Jorge Sequeira, Louise Christina Gonçalves Vasconcelos Siqueira, Hevila Rolim

**Affiliations:** 1 PROCISA, Universidade Federal de Roraima (UFRR), Av. Cap. Ene Garcez, 2413, Boa Vista, Roraima 69310-000 Brazil; 2 Instituto de Antropologia, Universidade Federal de Roraima, UFRR, Boa Vista, Roraima Brazil; 3 Universidade Federal de Roraima, UFRR, Boa Vista, Roraima Brazil; 4 Independent Researcher, Boa Vista, Roraima Brazil; 5 Universidade Federal de Rondônia, Porto Velho, Rondônia Brazil

**Keywords:** Yanomami, Glaucoma, Intraocular pressure, Cup-to-disc ratio, Fundoscopy, Indigenous health, Underserved populations, Amazon, Brazil

## Abstract

**Purpose:**

This study provides the first systematic characterisation of IOP, optic disc C/D ratio, and fundoscopic findings in the Yanomami, one of the largest and most geographically isolated Indigenous peoples of the Amazon, and identifies findings suggestive of glaucoma.

**Methods:**

Cross-sectional observational study conducted June–August 2024 at the Yanomami Indigenous Health House (CASAI-Y), Boa Vista, Brazil. One hundred fifty-eight self-identified Yanomami individuals aged  ≥ 5 years were examined. Non-contact tonometry (Huvitz HNT-7000), portable non-mydriatic fundus photography (Eyer), portable slit-lamp biomicroscopy, and direct ophthalmoscopy were performed. C/D ratio was graded by fundus photographic review. Associations were assessed by OR with 95% CI, Student t test, one-way ANOVA, Fisher exact test, and Spearman and Pearson correlation. *P* values were adjusted for multiple comparisons using the Benjamini–Hochberg procedure.

**Results:**

Among 151 participants with assessable IOP (secondary glaucoma case excluded from descriptive analysis), mean IOP was 13.64 mmHg (SD 1.59) OD and 13.93 mmHg (SD 1.75) OS; ocular hypertension was identified in 1 participant (0.66%). Mean C/D ratio was 0.261 (SD 0.129) OD and 0.267 (SD 0.138) OS among 148 participants; 3 (2.03%) had C/D  > 0.5 in at least one eye. No significant IOP–C/D correlation was found (Spearman rₛ = 0.105, *P* = .210 OD; rₛ = 0.058, *P* = .496 OS). Secondary angle-closure glaucoma was confirmed in one participant (IOP 35/55 mmHg, 360° synechiae, no light perception). Two further participants had findings suggestive of primary glaucoma. Ocular trauma was associated with retinal cicatricial lesions (OR 13.41; 95% CI 2.55–70.35; *P* = .008; P adj = .036) and, without retaining significance after adjustment for multiple comparisons, with fundoscopic abnormality (OR 7.98; 95% CI 1.26–50.55; *P* = .04; P adj = .10); both estimates rest on 7 trauma-exposed participants. No diabetic retinopathy was identified.

**Conclusions:**

This study provides the first quantitative ophthalmic baseline for the Yanomami regarding IOP, optic disc morphology, and fundoscopic findings. Primary open-angle glaucoma does not appear to constitute a relevant burden. Secondary glaucoma from complicated cataract is the most preventable severe presentation. Angle-closure glaucoma remains uncharacterised. Cataract surgical access, camerular angle screening, and glaucoma investigation are unmet priorities in this population.

## Key messages

**What is already known:**

Epidemiologic data on intraocular pressure (IOP), optic disc morphology, and glaucoma spectrum among remote Amazonian Indigenous peoples are virtually absent from the peer-reviewed literature. Prior studies in the Yanomami were limited to onchocerciasis-related ocular findings, with no cup-to-disc (C/D) ratio data and no investigation of primary angle-closure or secondary glaucoma.

**What this study adds:**

This is the first study to systematically characterise IOP, optic disc C/D ratio, and fundoscopic findings in the Yanomami people. Mean IOP (13.78 mmHg) and C/D ratio (0.261 OD) are uniformly low, with no significant IOP–C/D correlation, suggesting that primary open-angle glaucoma does not appear to constitute a relevant burden in this predominantly young cohort. Secondary glaucoma from hypermature cataract—directly preventable by timely surgical access—is the most severe finding. The burden of ocular trauma, never previously quantified in the Yanomami, warrants attention as a preventable cause of posterior segment abnormality, though the present association remains exploratory.

**Clinical implications:**

Expansion of cataract surgical access, camerular angle screening (gonioscopy and anterior segment OCT), and systematic glaucoma investigation are unmet priorities in the Yanomami population.

## Introduction

Glaucoma is the leading cause of irreversible blindness worldwide, affecting an estimated 80 million individuals in 2020, with projections exceeding 111 million by 2040 [1]. The disease encompasses a spectrum of optic neuropathies, with primary open-angle glaucoma (POAG) and primary angle-closure glaucoma (PACG) as the two dominant clinical entities, and secondary glaucomas accounting for a further relevant proportion of cases globally [2]. In low-resource settings, secondary angle-closure from complicated cataract is particularly relevant because it is directly preventable by timely surgical access.

Indigenous peoples of the Americas are among the most underserved populations in ophthalmology. Epidemiologic data on IOP, optic disc morphology, and posterior segment pathology among remote Amazonian groups are virtually absent from the peer-reviewed literature. The most directly relevant evidence for the Yanomami derives from two studies by Herzog-Neto and colleagues, which characterised ocular manifestations of onchocerciasis and their relationship to IOP [3, 4]. Those authors found that mean IOP among the Yanomami was lower than that of the general Brazilian population, that no cases of glaucoma were encountered, and that no structural damage to the optic nerve was identified, leading them to conclude that glaucoma did not constitute a relevant disease in this population.

A prior study from the same cohort at CASAI-Y characterised the burden of visual impairment and blindness in this population [5]. Fundoscopic abnormalities were the second leading cause of uncorrected visual impairment after cataract (22.22% of cases), motivating the present investigation. This study aimed to describe the distribution of IOP, optic disc C/D ratio, and fundoscopic findings in the Yanomami; to identify findings suggestive of glaucoma; and to identify factors associated with posterior segment abnormalities.

## Methods

### Study design and setting

Observational, cross-sectional, descriptive, and analytical study conducted June–August 2024 at CASAI-Y, Boa Vista, Roraima, Brazil—the sole regional referral facility of the Yanomami Special Indigenous Health District (DSEI-Y), serving Yanomami individuals temporarily relocated for health services unavailable at the primary care level. Geographic, legal, and logistical constraints preclude population-based surveys within the Yanomami Territory; CASAI-Y constitutes the most feasible and demographically representative access point. This study was performed in accordance with the tenets of the Declaration of Helsinki and was approved by the Research Ethics Committee of the Universidade Federal de Roraima and CONEP (No. 6.479.368-CEP/CONEP). Informed consent or assent was obtained from all participants. As an observational, cross-sectional descriptive study, it was not registered in a public trials registry.

### Participants

Self-identified Yanomami individuals aged 5 years or older present at CASAI-Y during data collection were eligible. Exclusion criteria were refusal to participate and inability to complete the examination owing to cognitive impairment or insufficient clinical conditions. Of 278 eligible individuals, 158 (56.8%) consented and were included; of the remaining 120, 118 refused participation and 2 were excluded owing to cognitive impairment or absence of minimal clinical conditions for examination [5]. Participants represented 23 of 37 base health centres across 6 of 7 macro-regions of the DSEI-Y, spanning all four principal Yanomami subethnic subgroups: Yanomami (n = 104, 65.8%), Sanuma (n = 38, 24.1%), Xirixana (n = 11, 7.0%), and Xiriana (n = 5, 3.2%). Mean age was 32.4 years (SD, 13.8); 102 (64.6%) were male.

### Procedures

Data collection followed a three-stage protocol. Stage 1: demographic data and informed consent with oral translation by native-speaking Indigenous interpreters; the consent process was audio-recorded given the high prevalence of illiteracy. Stage 2: objective measurements by trained technicians—IOP measured by a Huvitz HNT-7000 non-contact tonometer (Huvitz Co., Ltd; three readings per eye, highest value recorded); posterior segment imaging by an Eyer portable non-mydriatic fundus camera (Phelcom Technologies). Stage 3: comprehensive clinical examination by the principal investigator, including portable slit-lamp biomicroscopy and direct ophthalmoscopy. Optic disc C/D ratio was graded by fundus photographic review of images captured in Stage 2 (decimal fraction, 0.1–1.0 per eye) and estimated as the ratio of the vertical cup diameter to the vertical disc diameter, with the disc margin defined by the inner edge of the scleral ring and the cup margin by the point of neuroretinal rim inflexion, using a standardised 0.1 decimal scale. All images were graded by a single ophthalmologist.

Ocular hypertension was defined as IOP greater than 21 mmHg. Glaucoma suspicion was defined as C/D greater than 0.6, inter-eye C/D asymmetry greater than 0.2, or IOP greater than 21 mmHg, following criteria applied in Brazilian glaucoma services [22]. Because camerular angle assessment, automated perimetry, and OCT were not available in this field setting, formal glaucoma diagnosis according to current criteria [6] could not be established; findings are reported as presentations suggestive of glaucoma, requiring confirmatory workup. Participants with findings requiring follow-up were referred via the Unified Health System (SUS) regulatory service.

### Statistical analysis

Continuous variables are presented as means and SDs. Categorical variables are presented as frequencies and percentages. Comparisons by sex used independent-samples Student t test; comparisons across age groups and subethnic subgroups used one-way ANOVA. Spearman rank correlation (rₛ) was used to assess IOP–C/D relationships, given the ordinal nature of the C/D ratio grading; Pearson correlation (r) was used to assess the IOP–age relationship, as both variables were continuous. Categorical associations were assessed by chi-square or Fisher exact test, with ORs and 95% CIs. All tests were two-sided; *P* < 0.05 was considered statistically significant. Because multiple comparisons were performed, *P* values were adjusted for the false discovery rate using the Benjamini–Hochberg procedure across the 14 tests reported (subgroup comparisons for IOP, C/D ratio, and fundoscopic findings; the IOP–age and IOP–C/D correlations; and the two trauma associations). Unadjusted *P* values are reported throughout; adjusted values (P adj) are given for findings reaching nominal significance. Statistical analyses were performed with Microsoft Excel, Statistica 12 (TIBCO Software Inc.), and StatPlus (AnalystSoft).

## Results

### Participation and sample representativeness

Of 278 eligible individuals, 158 (56.8%) consented to participate. Males participated at a substantially higher rate than females (71.8% vs. 41.2%; OR, 3.64; 95% CI, 2.20–6.00; *P* < 0.001), with statistically significant sex differences observed across the 0–19, 20–39, and 40–59 year age groups [5]. Participation rates varied across subethnic subgroups: the Yanomami subgroup showed higher acceptance compared to other subgroups (OR, 2.89; 95% CI, 1.69–4.96; *P* < 0.001), whereas the Sanumã subgroup showed markedly lower participation (OR, 0.39; 95% CI, 0.24–0.64; *P* < 0.001) [5]. A moderate and statistically significant correlation was observed between participant distribution and total community population (r = 0.57; *P* = 0.0002), strengthening when restricted to the eligible age group (r = 0.75; *P* < 0.001) [5], supporting the demographic alignment of the sample with the broader Yanomami population.

### Intraocular pressure

**Fig. 1:**
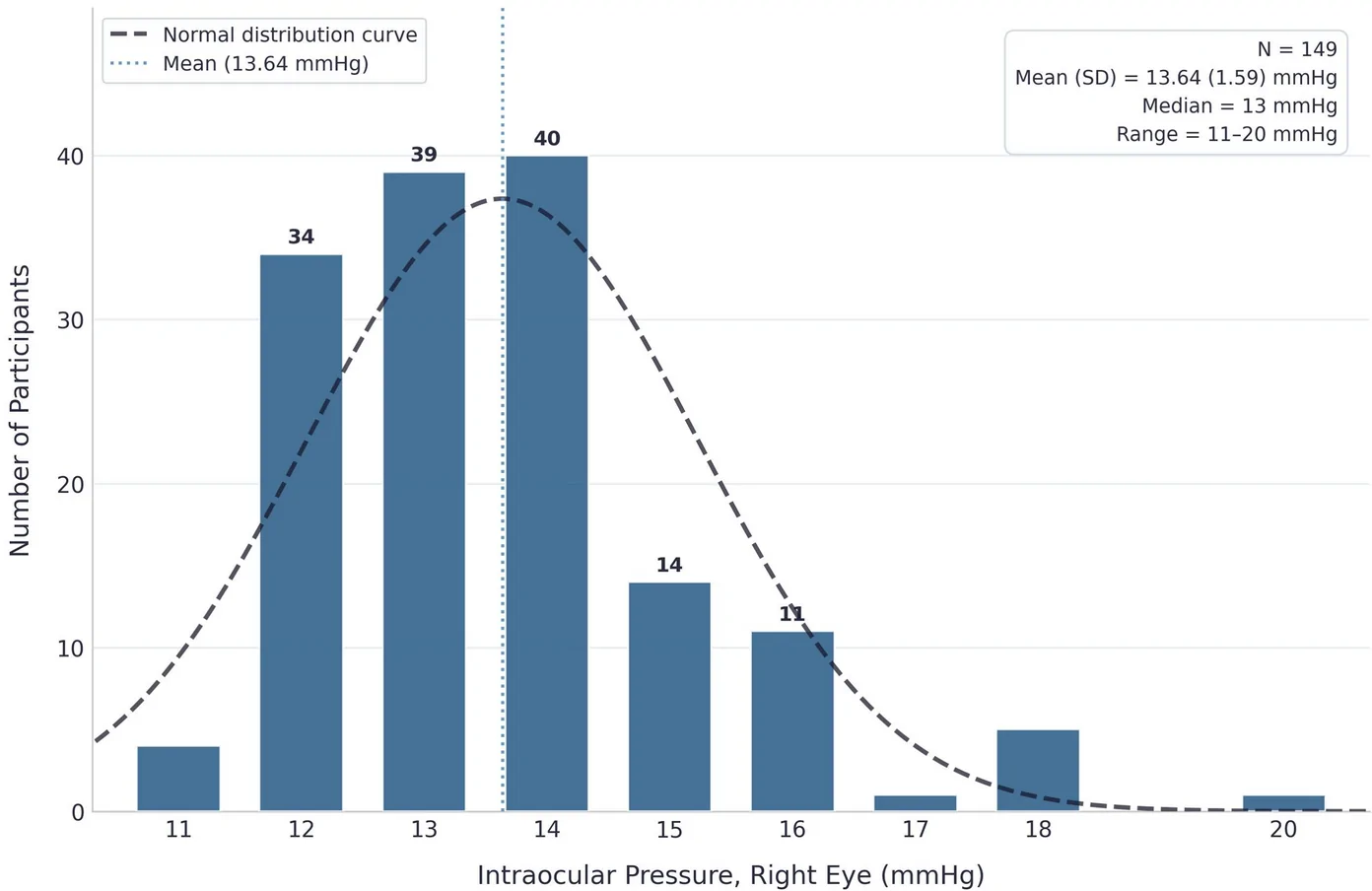
Distribution of intraocular pressure (IOP) in the right eye (OD) among Yanomami participants (N = 149; secondary glaucoma case excluded). Bars represent the number of participants per 1-mmHg interval. The dashed line represents the fitted normal distribution curve. The dotted vertical line marks the sample mean, 13.64 mmHg (SD, 1.59); median, 13 mmHg; range 11–20 mmHg

Of 158 participants, IOP could not be assessed in 6: three children did not cooperate with the non-contact tonometer, one adult had severe motor impairment, and two had conditions rendering measurement technically impossible (phthisis bulbi and anophthalmia). IOP was obtained in 152 participants; the confirmed secondary glaucoma case (IOP 35 mmHg OD and 55 mmHg OS) was excluded from descriptive analyses, yielding an analytical sample of 151 participants and 299 valid measurements. Mean IOP was 13.64 mmHg (SD, 1.59) OD and 13.93 mmHg (SD, 1.75) OS; overall mean was 13.78 mmHg (SD, 1.67). The distribution was concentrated in the 12–15 mmHg range, with a modal value of 14 mmHg (Fig. 1). Ocular hypertension (IOP  > 21 mmHg) was identified in 1 participant (0.66%), corresponding to a single eye with an IOP of 22 mmHg. IOP did not differ significantly by sex (t =  − 0.117; *P* = 0.907) or subethnic subgroup (F = 0.545; *P* = 0.652). Mean IOP differed across age groups (F = 3.688; *P* = 0.012; P adj = 0.043), rising from 13.64 mmHg in the 0–19 group to 14.19 mmHg in the 40–59 group, and a weak positive correlation was found between IOP and age (r = 0.168; *P* = 0.004; P adj = 0.025). Distributions are presented in Table 1.

**Table 1:** Distribution of intraocular pressure (IOP) by sex, age group, and subethnic subgroup (N = 151)

	No	IOP OD, Mean (SD), mmHg	IOP OS, Mean (SD), mmHg	*P* Value
*Sex*				
Female	52	13.63 (1.90)	13.90 (1.82)	
Male	99	13.64 (1.42)	13.94 (1.72)	.907ᵁ
*Age group, y*				
0–19	29	13.52 (1.55)	13.76 (1.41)	
20–39	65	13.47 (1.56)	13.52 (1.68)	
40–59	55	13.85 (1.64)	14.52 (1.88)	
60–80	2	15.00 (1.41)	13.50 (0.71)	.012ᵇ
*Subethnic subgroup*				
Yanomami	97	13.74 (1.72)	13.95 (1.70)	
Sanuma	38	13.50 (1.18)	13.97 (1.88)	
Xirixana	11	13.20 (1.03)	14.09 (1.58)	
Xiriana	5	13.60 (2.61)	12.80 (2.17)	.652ᵇ
**Total**	**151**	**13.64 (1.59)**	**13.93 (1.75)**	

Bold values indicate the total (overall sample) values.

IOP, intraocular pressure; OD, right eye; OS, left eye; SD, standard deviation. ᵁIndependent-samples t test (sex comparison). ᵇOne-way ANOVA (age group and subethnic subgroup comparisons). The 60–80 year stratum comprises 2 participants (4 eyes) and is reported for descriptive completeness only; comparisons involving this stratum are unstable. Collapsing this stratum with the 40–59 group did not alter the findings (F = 5.554, *P* = .004). IOP values of the confirmed secondary glaucoma case (IOP, 35/55 mmHg) excluded from descriptive analyses

### Optic disc cup-to-disc ratio

C/D ratio was assessed in 148 participants with viable optic disc images in at least one eye (Table 2), yielding 147 valid OD and 144 valid OS measurements. Mean C/D was 0.261 (SD, 0.129) OD and 0.267 (SD, 0.138) OS, with a modal value of 0.2 (OD, 32.4%; OS, 34.0%). C/D greater than 0.5 was found in 2 OD (1.4%) and 3 OS (2.1%); considering at least one eye, 3 of 148 participants (2.03%) met this threshold. Marked inter-eye C/D asymmetry (> 0.2) was identified in 1 of 144 participants with both eyes assessable (0.69%). C/D ratio did not differ significantly by sex (t =  − 0.993, *P* = 0.321), age group (F = 0.066, *P* = 0.978), or subethnic subgroup (F = 0.310, *P* = 0.818). No significant IOP–C/D correlation was found (OD: rₛ = 0.105, *P* = 0.210; OS: rₛ = 0.058, *P* = 0.496).

**Table 2:** Distribution of optic disc C/D ratio by sex, age group, and subethnic subgroup (N = 148)

	No	C/D OD, Mean (SD)	C/D OS, Mean (SD)	*P* Value
*Sex*				
Female	53	0.251 (0.117)	0.257 (0.115)	
Male	95	0.266 (0.136)	0.274 (0.150)	.321ᵁ
*Age group, y*				
0–19	30	0.273 (0.117)	0.267 (0.121)	
20–39	61	0.266 (0.140)	0.262 (0.137)	
40–59	55	0.247 (0.125)	0.277 (0.152)	
60–80	2	0.300 (0.141)	0.200 (0.000)	.978ᵇ
*Subethnic subgroup*				
Yanomami	98	0.258 (0.128)	0.266 (0.141)	
Sanuma	35	0.260 (0.122)	0.263 (0.129)	
Xirixana	10	0.270 (0.142)	0.280 (0.140)	
Xiriana	5	0.300 (0.200)	0.300 (0.158)	.818ᵇ
**Total**	**148**	**0.261 (0.129)**	**0.267 (0.138)**	

Bold values indicate the total (overall sample) values.

C/D, cup-to-disc ratio; OD, right eye; OS, left eye; SD, standard deviation. ᵁIndependent-samples t test. ᵇOne-way ANOVA. Only participants with viable optic disc images in at least one eye included. The 60–80 year stratum comprises 2 participants (4 eyes) and is reported for descriptive completeness only; comparisons involving this stratum are unstable. Collapsing this stratum with the 40–59 group did not alter the findings (F = 0.083, *P* = .921)

### Findings suggestive of glaucoma

Three participants had clinically relevant findings. Case 1—advanced optic neuropathy, normotensive phenotype. A 41-year-old male presented with pain in the left eye and a lifelong history of severe visual impairment. C/D ratio was 0.2 OD and 0.9 OS with altered vascular calibre in both eyes, and IOP was 18 mmHg OD and 13 mmHg OS. Presenting visual acuity was 20/400 OD and counting fingers OS; after best optical correction, visual acuity improved to 20/100 OD while the left eye remained unchanged, indicating irreversible visual loss. Case 2—secondary glaucoma from complicated cataract. A 49-year-old female presented with total bilateral cataract, bilateral paralytic mydriasis, and 360° posterior synechiae, consistent with hypermature cataract complicated by angle-closure glaucoma with pupillary block. IOP was 35 mmHg OD and 55 mmHg OS, with no light perception bilaterally. Optic disc assessment was not feasible due to complete media opacity. This presentation is attributable to delayed surgical access, and secondary glaucoma was confirmed on clinical grounds alone. Case 3—glaucoma suspect, elevated bilateral C/D ratio. A 45-year-old female presented with C/D 0.6 bilaterally, IOP 20 mmHg OD and 19 mmHg OS, normal biomicroscopy, and preserved visual acuity. This case requires confirmatory investigation with camerular angle assessment (gonioscopy and anterior segment OCT), automated perimetry, and OCT.

### Fundoscopic findings

Fundoscopy was evaluable in 291 of 316 eyes (92.1%). Normal bilateral fundoscopy was present in 119 of 158 participants (75.3%; Table 3). Of 291 evaluated eyes, 13 (4.45%) had retinal cicatricial lesions and 28 (9.62%) had macular reflex alterations. No diabetic retinopathy, retinal haemorrhage, or exudates were identified.

**Table 3:** Fundoscopic classification of participants by sex, age group, and subethnic subgroup (N = 158)

	No	Normal Bilateral, No. (%)	Normal/Altered, No. (%)	Bilateral Altered, No. (%)	Unavailable, No. (%)	Other, No. (%)
*Sex*						
Female	56	47 (83.9)	4 (7.1)	2 (3.6)	3 (5.4)	0 (0.0)
Male	102	72 (70.6)	13 (12.7)	5 (4.9)	7 (6.9)	5 (4.9)
*Age group, y*						
0–19	32	29 (90.6)	1 (3.1)	0 (0.0)	2 (6.2)	0 (0.0)
20–39	67	55 (82.1)	3 (4.5)	2 (3.0)	6 (9.0)	1 (1.5)
40–59	57	34 (59.6)	13 (22.8)	4 (7.0)	2 (3.5)	4 (7.0)
60–80	2	1 (50.0)	0 (0.0)	1 (50.0)	0 (0.0)	0 (0.0)
*Subethnic subgroup*						
Yanomami	104	81 (77.9)	12 (11.5)	4 (3.8)	6 (5.8)	1 (1.0)
Sanuma	38	27 (71.1)	1 (2.6)	3 (7.9)	3 (7.9)	4 (10.5)
Xirixana	11	7 (63.6)	3 (27.3)	0 (0.0)	1 (9.1)	0 (0.0)
Xiriana	5	4 (80.0)	1 (20.0)	0 (0.0)	0 (0.0)	0 (0.0)
**Total**	**158**	**119 (75.3)**	**17 (10.8)**	**7 (4.4)**	**10 (6.3)**	**5 (3.2)**

Bold values indicate the total (overall sample) values.

Normal/altered indicates one eye normal and one eye with any alteration. Unavailable indicates both eyes inviable (opaque media). Other indicates one eye normal and one eye inviable. Classification is per participant

A history of ocular trauma was associated with fundoscopic abnormality (OR, 7.98; 95% CI, 1.26 to 50.55; *P* = 0.04), although this association did not retain significance after adjustment for multiple comparisons (P adj = 0.10), and with retinal cicatricial lesions (OR, 13.41; 95% CI, 2.55 to 70.35; *P* = 0.008; P adj = 0.036; Table 4). The wide confidence intervals reflect the small number of participants with a trauma history (n = 7). Fundoscopic abnormality was more frequent in participants aged  ≥ 40 years than in younger participants (31.6% vs. 6.6%; OR, 6.54; 95% CI, 2.41 to 17.75; *P* < 0.001; P adj = 0.002). No significant associations were found with sex (*P* = 0.33) or subethnic subgroup (*P* = 0.56).

**Table 4:** Association between ocular trauma and fundoscopic findings

	Normal/Absent, No. (%)	Altered/Present, No. (%)	Total, No	Altered/Present, %	OR (95% CI); *P* Value
*Fundoscopic abnormality (n* = *144)*					
Ocular trauma, no	117 (84.2)	22 (15.8)	139	15.8	Reference
Ocular trauma, yes	2 (40.0)	3 (60.0)	5	60.0	7.98 (1.26 to 50.55); .04
*Retinal cicatricial lesion (n* = *158)*					
Ocular trauma, no	143 (94.7)	8 (5.3)	151	5.3	Reference
Ocular trauma, yes	4 (57.1)	3 (42.9)	7	42.9	13.41 (2.55 to 70.35); .008

CI, confidence interval; OR, odds ratio. Fisher exact test used throughout. The fundoscopic abnormality analysis is restricted to the 144 participants with an assessable fundus (14 participants with inviable media excluded), of whom 5 reported ocular trauma. The retinal cicatricial lesion analysis includes all 158 participants; lesion indicates presence in at least one eye, and participants whose fundus could not be assessed were conservatively classified as having no lesion. The wide confidence intervals reflect the small number of participants with a history of ocular trauma (n = 7); these associations should be interpreted with caution

## Discussion

This study provides the first systematic characterisation of IOP, optic disc morphology, and fundoscopic findings in the Yanomami. The principal findings are: IOP is uniformly low (overall mean, 13.78 mmHg) without significant variation by demographic group; mean C/D ratio is markedly low (0.261 OD); no significant IOP–C/D correlation exists; POAG does not appear to constitute a prevalent pathology. Secondary glaucoma from complicated cataract was confirmed in one participant, resulting in bilateral blindness attributable to delayed diagnosis and care; advanced optic neuropathy of undetermined aetiology was identified in a second participant requiring confirmatory diagnostic investigation; and a third participant is a glaucoma suspect based on elevated bilateral C/D ratio alone. Ocular trauma showed an exploratory association with posterior segment abnormality, limited by the small trauma-exposed sample.

### Intraocular pressure and open-angle glaucoma

The mean IOP of 13.78 mmHg is consistent with prior findings from a Yanomami onchocerciasis-endemic community, where Herzog-Neto et al. reported a mean IOP of 10.47 mmHg [4], and with values described in other isolated Indigenous populations in whom primary open-angle glaucoma was absent (mean IOP 11.7–12.0 mmHg among Alaskan Eskimos) [15]. Ocular hypertension was identified in a single participant (0.66%), compared with a prevalence of 2.1% (95% CI, 1.4 to 2.8) reported in a population-based survey in South Brazil using Goldmann applanation tonometry, which also documented POAG in 2.4% and PACG in 0.7% of individuals older than 40 years [7]; the younger age structure of the present sample precludes direct comparison. IOP increased weakly with age (r = 0.168; *P* = 0.004), with a difference of approximately 0.7 mmHg between the youngest and oldest strata. This gradient is of small magnitude and without apparent clinical consequence: mean IOP remained well below the ocular hypertension threshold in every age stratum. Notably, blood pressure in isolated Yanomami villages shows no age-related rise across the lifespan (age–systolic blood pressure slope, 0.00 mmHg per year; *P* = 0.98) [8]; the modest IOP–age gradient observed here therefore does not parallel systemic blood pressure trends in this population. The prevalence of C/D greater than 0.5 in at least one eye (3 of 148 participants; 2.03%) is lower than the 6.5% prevalence of glaucomatous cupping reported among older adults with normal visual acuity in the Brazilian Amazon Region [20], although that population was substantially older than the present sample and the comparison should therefore be interpreted with caution. The mean C/D ratio of 0.261 observed here is far below the mean values reported in glaucoma referral populations—0.69 (SD, 0.16) OD and 0.68 (SD, 0.16) OS among 1484 patients at a Brazilian glaucoma service [22], and a mean vertical C/D of 0.76 in a clinical series of patients with confirmed primary open-angle glaucoma [9]—indicating that optic disc morphology in this cohort lies well outside the glaucomatous range. The absence of IOP–C/D correlation supports the hypothesis that existing cupping is unlikely to reflect IOP-driven glaucomatous neuropathy, consistent with the low POAG prevalence reported in other Brazilian Indigenous communities [10, 11]; an ophthalmic survey of 193 Kadiwéu Indigenous people likewise identified no cases of ocular hypertension or glaucoma [17].

### Primary angle-closure glaucoma: an uncharacterised risk

Although camerular angle assessment was not performed, the risk of PACG warrants explicit consideration. In Caucasian populations, POAG accounts for 75–95% of primary glaucoma; however, in Inuit and certain Amerindian populations, PACG constitutes a substantially higher proportion [12]. In Greenland Inuit, PACG prevalence in individuals older than 40 years has been reported at 1.6–5.1%, with shallow anterior chamber depth as the primary anatomical risk factor [13, 14]. Alaskan Native populations similarly showed PACG as the predominant glaucoma subtype, particularly in women older than 60 years [15]. Acute primary angle closure is a vision-threatening ophthalmic emergency in which favourable outcomes depend on timely and appropriate intervention [21]; in a population for whom specialised ophthalmic care requires transfer from remote territory to Boa Vista, such an event would carry a substantial risk of irreversible visual loss. The systematic absence of camerular angle assessment from all prior Yanomami ocular health studies means that PACG prevalence remains entirely unknown—a critical gap that future studies should address by incorporating gonioscopy and anterior segment OCT [16].

### Secondary glaucoma and complicated cataract

The most clinically severe finding—bilateral IOP greater than 35 mmHg, total cataract, paralytic mydriasis, and 360° synechiae—illustrates secondary angle-closure glaucoma from hypermature cataract with pupillary block, directly preventable by timely surgical access. The Yanomami have a documented cataract prevalence of 7.59% at CASAI-Y [5], and geographic isolation, SUS referral delays, and cultural barriers all contribute to prolonged surgical delay. These findings provide empirical support for the urgent expansion of mobile surgical capacity and teleophthalmology-assisted triage within the DSEI-Y.

### Fundoscopic findings and ocular trauma

The association between ocular trauma and posterior segment abnormality has not been previously quantified in this population. This finding is exploratory: the small number of participants with a trauma history (n = 7) yields wide confidence intervals (OR, 7.98; 95% CI, 1.26 to 50.55 for fundoscopic abnormality; OR, 13.41; 95% CI, 2.55 to 70.35 for retinal cicatricial lesions), and the estimates should therefore be interpreted with caution. Nonetheless, the signal is clinically coherent: ocular trauma is a recognised cause of ocular complications, including secondary glaucoma. Cicatricial retinal lesions present an aetiologic differential including post-traumatic scarring, sequelae of onchocercal chorioretinitis [3, 18], and toxoplasmosis. The absence of diabetic retinopathy is consistent with the preserved traditional metabolic profile of the Yanomami and provides a public health baseline for monitoring future lifestyle transitions [19].

### Participation, representativeness, and generalisability

The lower participation rate among women (41.2% vs. 71.8% among men) warrants careful interpretation. Within Yanomami communities, women’s engagement in health research is commonly shaped by caregiving responsibilities, restrictions on mobility, and the cultural practice of seeking partner authorisation before interacting with non-Indigenous individuals [5]. These dynamics are consistent with broader patterns of structural barriers to healthcare participation reported across Indigenous populations in the Americas [11]. Although these sociocultural factors do not invalidate the findings, they may limit the precision of sex-stratified estimates. Regarding geographic representativeness, the moderate to strong correlations between participant distribution and Yanomami population distribution across base health centres (r = 0.57 to 0.75; *P* < 0.001) [5] support the potential to extrapolate the findings to the general Yanomami population, while acknowledging that individuals in the most remote communities may remain underrepresented.

### Limitations

The cross-sectional design precludes causal inference. The cohort has a young age structure, with a median age of 31 years and only 2 participants aged 60 years or older. The 40–59 year stratum, in which primary open-angle glaucoma begins to manifest, was nonetheless well represented (n = 56) and showed no signal: mean IOP was 14.19 mmHg with a single eye above 21 mmHg, and mean C/D ratio was 0.262, with 2 participants having C/D greater than 0.5 in at least one eye. The absence of a POAG burden is therefore not merely an artefact of the young sample. The stratum aged 60 years or older is, however, minimally represented, and the finding should be understood as conditional on this age distribution rather than as evidence of a protective effect. Dedicated assessment of older Yanomami individuals, in whom age-related glaucomatous change would be expected to emerge, is a priority for future investigation. The lower female participation rate (41.2% vs. 71.8% among males; OR, 3.64; 95% CI, 2.20 to 6.00) may have introduced selection bias in sex-stratified analyses [5]. The eligible population was itself sex-balanced (48.9% female), and the barriers described for women in this setting—caregiving responsibilities, restricted mobility, and the need for partner authorization—relate to social organisation rather than to ocular health status [5], suggesting that selection was probably non-differential with respect to the outcomes assessed here. Consistent with this, IOP, C/D ratio, and fundoscopic findings did not differ by sex (all *P* > 0.30). The smaller female sample nevertheless reduces the precision of sex-specific estimates, and residual unmeasured selection effects cannot be excluded. The absence of camerular angle assessment prevents angle characterisation and formal PACG diagnosis—the most clinically significant methodological limitation. IOP was measured by non-contact tonometry without corneal pachymetry and was recorded as the highest of three readings rather than the mean, which may overestimate absolute IOP; however, as values were uniformly low even under this conservative approach, this is unlikely to have affected the study’s conclusions. C/D ratio was graded by a single examiner from non-mydriatic, non-stereoscopic fundus photographs, without OCT confirmation; no blinded second grading was performed, and inter- or intra-rater reliability could not be assessed. C/D estimates should therefore be interpreted with this constraint in mind. Automated perimetry was unavailable; formal primary glaucoma diagnosis could not be established in two of the three suspected cases, which require confirmatory investigation with camerular angle assessment (gonioscopy and anterior segment OCT), automated perimetry, and OCT. Family history and genetic data were not collected. The Yanomami are a population of recent contact, for whom documented genealogies and prior genetic studies are unavailable, and for whom systematic collection of family history is constrained by linguistic and cultural barriers. A hereditary contribution to the bilateral cataract observed in a 49-year-old participant therefore cannot be assessed. This case—the most severe finding in the cohort—indicates the need for dedicated investigation of glaucoma as well as related hereditary ocular disease in this population. Multiple comparisons were performed; *P* values were adjusted using the Benjamini–Hochberg procedure, and the age-related associations for IOP and for fundoscopic abnormality, as well as the association between trauma and retinal cicatricial lesions, retained significance after adjustment. The association between trauma and fundoscopic abnormality did not, and is reported as exploratory.

## Conclusions

This study provides the first quantitative ophthalmic baseline for the Yanomami regarding IOP, optic disc morphology, and fundoscopic findings. POAG does not appear to constitute a relevant burden. Secondary glaucoma from complicated cataract is the most severe and preventable finding, directly attributable to surgical inaccessibility. Angle-closure glaucoma remains entirely uncharacterised and requires dedicated investigation incorporating gonioscopy and anterior segment OCT. Ocular trauma, not previously quantified in this population, warrants further investigation as a preventable cause of posterior segment abnormality, complementing complicated cataract as a target for prevention in this population. Integration of glaucoma assessment and expanded cataract surgical access are unmet priorities for Indigenous eye health in Brazil.

## Abbreviations

CASAI-Y: Yanomami Indigenous Health House
C/D: Cup-to-disc ratio
DSEI-Y: Yanomami Special Indigenous Health District
IOP: Intraocular pressure
OCT: Optical coherence tomography
OR: Odds ratio
PACG: Primary angle-closure glaucoma
POAG: Primary open-angle glaucoma
SD: Standard deviation
SUS: Brazilian Unified Health System

## Declarations

### Data sharing

Individual participant data cannot be shared without prior consultation and formal authorisation from the Yanomami communities involved, pursuant to Indigenous community governance requirements and ethical oversight by CONEP. Requests may be addressed to the corresponding author and will be reviewed individually, subject to community approval.

### Generative AI declaration

During preparation of this work, the authors used Claude (Anthropic, San Francisco, CA, USA) to support language editing and manuscript structuring in English, and to assist in re-checking statistical computations against the primary dataset. All study design, analytical decisions, interpretation, and final validation of results were performed by the authors. After using this tool, the authors reviewed and edited all content and take full responsibility for the integrity and accuracy of the final version.
